# Prevalence and molecular basis of glucose-6-phosphate dehydrogenase deficiency in Afghan populations: implications for treatment policy in the region

**DOI:** 10.1186/1475-2875-12-230

**Published:** 2013-07-08

**Authors:** Toby Leslie, Bushra Moiz, Nader Mohammad, Omar Amanzai, Haroon ur Rasheed, Sakhi Jan, Abdul M Siddiqi, Amna Nasir, Mohammad A Beg, Martijn Vink

**Affiliations:** 1 HealthNet TPO, Kabul, Afghanistan; 2 London School of Hygiene and Tropical Medicine, London, UK; 3 Aga Khan University, Karachi, Pakistan; 4 HealthNet TPO, Amsterdam, The Netherlands

**Keywords:** Malaria, G6PD, Glucose-6-phosphate dehydrogenase deficiency, Afghanistan, Primaquine, Vivax, Relapse

## Abstract

**Background:**

Glucose-6-phosphate dehydrogenase deficiency (G6PD), an x-linked inherited enzymopathy, is a barrier to malaria control because primaquine cannot be readily applied for radical cure in individuals with the condition. In endemic areas, including in Afghanistan, the G6PD status of vivax patients is not routinely determined so the drug is rarely, if ever, prescribed even though it is included as a recommended treatment in local, regional and global guidelines. This study assessed the prevalence and genotype of G6PD deficiency in Afghan populations and examined the need for routine G6PD testing as a malaria treatment and control tool.

**Methods:**

A cross-sectional household survey was conducted using random sampling in five Afghan cities to determine the prevalence of G6PD deficiency in Afghan ethnic groups. Filter-paper blood spots were analysed for phenotypic G6PD deficiency using a fluorescent spot test. Molecular analysis was conducted to identify the genetic basis of the disorder.

**Results:**

Overall, 45/1,436 (3.1%) people were G6PD deficient, 36/728 (5.0%) amongst males and 9/708 (1.3%) amongst females. Amongst males the prevalence was highest in the Pashtun ethnic group (10%, 26/260) while in Tajik males it was 8/250 (3.2%); in Hazara males it was 1/77 (1.3%) and in Uzbek males is was 0/125. Genetic testing in those with deficiency showed that all were of the Mediterranean type (Med-) characterized by a C-T change at codon 563 of the G6PD gene.

**Conclusion:**

Prevalence of G6PD deficiency in Afghanistan varies considerably by ethnic group and is predominantly of the Mediterranean type. G6PD deficient individuals are susceptible to potentially severe and life-threatening haemolysis after standard primaquine treatment. If the aim of increasing access to radical treatment of vivax is to be successful reliable G6PD testing needs to be made routinely available within the health system.

## Background

Glucose-6-phosphate dehydrogenase (G6PD) deficiency is the most common genetic enzyme disorder in humans [1]. Worldwide, approximately 400 million people carry the deficiency gene and over 140 G6PD variants have been genetically characterized. The worldwide distribution closely matches that of malaria and provides some protection, suggesting that the disease has exerted evolutionary pressure for retention of the trait in humans [2]. Because the G6PD gene is located on the X-chromosome, the prevalence of G6PD in males is higher than that of females [1]. The deficiency can cause haemolytic episodes ranging from subclinical and mild to severe and fatal in response to stressors which include commonly used drugs. The G6PD deficiency occurring in ~10% of men of African descent (African (A-) variant) is regarded as milder than other forms although fatal reactions to primaquine have been recorded in a limited number of patients [3]. The Mediterranean variant (Med-) is regarded as more severe.

Although commonly asymptomatic, G6PD-deficiency becomes clinically relevant in response to oxidative stress [4] including from the anti-malarial drug, primaquine [5]. Red cells of those with severe deficiency are susceptible to haemolysis when exposed to therapeutic doses of the drug.

Primaquine is the only licensed medicine for treatment of the dormant liver stage of *Plasmodium vivax*. The parasite’s ability to form latent hynozoites in infected individuals causes relapse episodes for months or years following the initial infection without the bite of an infective mosquito. The hynozoite is therefore responsible for a large proportion of vivax malaria cases and is the principle disease reservoir.

In Afghanistan, as in most of Asia, malaria is caused predominantly by *P. vivax* but the unknown G6PD status in most patients guarantees that almost none receive adequate drug therapy for radical cure of confirmed vivax malaria. This situation is similar in most areas of south Asia, where G6PD testing is not routinely applied, representing a major impediment to control of the disease and a failure of policy over practice.

No systematic estimates of G6PD deficiency prevalence exists for Afghanistan. In order to inform decision making on the need for G6PD testing in the health system, a cross-sectional study was undertaken to estimate the prevalence of G6PD deficiency in the population and to characterize the G6PD deficiency variants.

## Methods

The primary objective of the study was to estimate the prevalence of G6PD deficiency in the population of Afghanistan. The secondary objectives were to estimate the prevalence of G6PD deficiency among different ethnics groups in Afghanistan and to identify the G6PD deficiency variants in the population.

### Sample selection

A cross-sectional study design was used encompassing five major Afghan cities. Afghanistan is ethnographically diverse and the study settings were applied to reflect this diversity. The most populous ethnic groups are Pashtun and Tajik with fewer from Hazara, Uzbek and Turkmen groups and small minorities of Pashaei, Noristani, Arab, and mixed ethnic groups

The study was conducted in major urban centres of five Afghan provinces: Nangarhar (Jalalabad), Takhar (Taloquan), Faryab (Maimana), Bamyan (Bamyan) and Kabul (Kabul City).

Nangarhar is located in the east of the country and has the highest intensity malaria transmission of the study sites (ten to 100 cases per 1,000 persons per year). The province is dominated by Pashtun ethnicity, but two minority ethnic groups, Arabs and Pashaees, are also settled in Nangarhar. The north-eastern province of Takhar is endemic for malaria, but at lower levels than in the east and has predominantly Tajik and Uzbek ethnicity. The north-western province of Faryab, endemic for malaria with very low transmission levels (<1/1,000 per year) and contains Turkmen, Uzbek and Tajik ethnic groups. Bamyan’s population in the Central Highlands of the country is predominantly of Hazara ethnic group, and Kabul is the multi-ethnic capital city of the country with all groups represented. Neither Bamyan nor Kabul is endemic for malaria.

The sample sites, therefore, reflect the majority of ethnic groups in Afghanistan and are readily accessible in terms of security, geography and resource availability. The sample was not intended to (and does not) estimate the relative proportion of each ethnic group in the population as a whole.

To detect a prevalence of G6PD deficiency >3% in the population [6], with 90% power at the 95% confidence level, 942 persons were required. To establish reasonably precise estimates within individual ethnic groups and to account for differences by gender required a larger sample size of 1,500. The sample collected from each city was proportionate to relative populations (Table 1).

**Table 1:** Population and proportion in the sample

	Total*	**% of total**	**Number in study**
Jalalabad	1,289,000	20	297
Kabul	3,138,000	48	722
Maimana	858,000	13	197
Taloquan	845,000	13	194
Bamyan	387,000	6	89
TOTAL	6,517,000	100	1,500

* Data from Central Statistics Office 1385 (2007).

Households were selected using random transect lines drawn within the municipal limits on maps of each city [7]. Each transect was used to identify households, selecting every third house and then every eighth, 13^th^, etc. house (using a sampling interval of five), until the required number of households were recruited. Two persons per household (one male and one female) were selected. Additional transects were used in the same way if too few houses were available on the initial transect.

One male and one female volunteer who matched the inclusion and none of the exclusion criteria were selected. The sample included any ethnic group living in the study household; males and females; and those aged over three years. Participants were excluded if they refused to give consent; were under three years of age; were incompetent to give consent; or were infirm. A simple pretested questionnaire was used for data collection. Informed consent was taken at enrolment into the study. Ethical approval was given by the Institutional Review Board of the Ministry of Public Health of Afghanistan.

### Detection of G6PD deficiency

Trained surveyors collected filter paper blood spots (Whatman 3M filter papers) marked with the patient number and stored in sealed bags with silica beads. Samples were processed on the following day.

**Figure 1:**
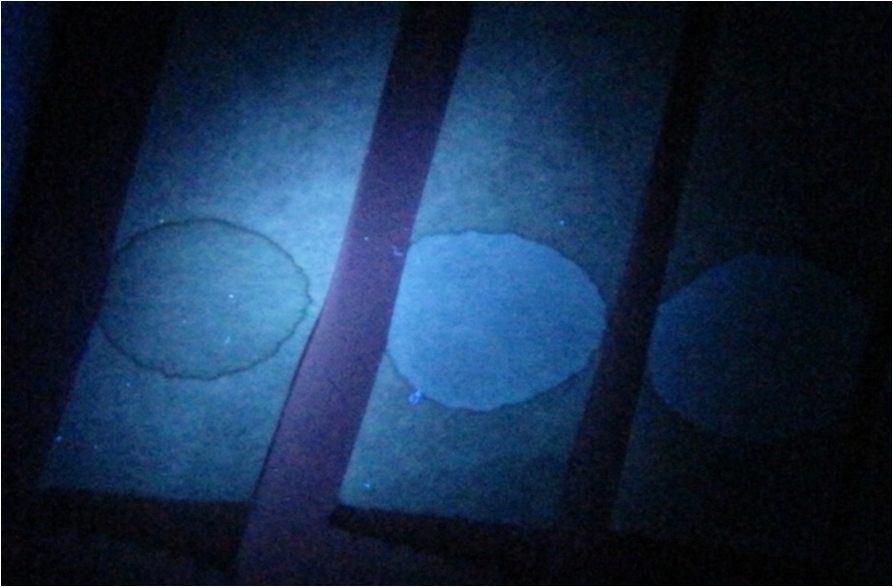
**G6PD fluorescent spot test under ultraviolet light (365nm) (Beutler test) [**[8]**].** The left filter paper shows severe deficiency, and the two on the right show a normal reaction.

G6PD deficiency status was based on a fluorescent spot test (FST) (PishtazTeb Diagnostics, Tehran, Islamic Republic of Iran) to identify the production of nicotinamide adenine dinucleotide phosphate-oxidase (NADPH) from nicotinamide adenine dinucleotide phosphate (NADP) [8]. The FST is a standard qualitative test for the detection of G6PD deficiency and can be performed in about 15 min. The test is considered positive (i.e. a patient is deficient) when the blood spot fails to fluoresce under ultraviolet light (Figure 1).

Genetic analysis of the samples was undertaken at The Aga Khan University Hospital, Karachi, Pakistan using the filter paper blood-spots collected in the field. Due to resource constraints, a subsample was selected to include all samples with detected deficiency and, by gender, 10% of negative samples from male participants and 50% of all female samples. G6PD non-deficient subjects were included to confirm the G6PD screening test and to identify the proportion of females carrying heterozygous copies of the gene, which are not detected by the fluorescent spot test.

Genomic DNA was extracted from dried blood spots using mini Qiagen® DNA Isolation kit (Qiagen Inc, Chatsworth, CA, USA) according to the manufacturer’s instructions. DNA was eluted in 50 ul of elution buffer and stored at −20°C until use.

Subsequently the DNA was employed as a template in a PCR reaction which used two gene specific primers and the reagents required for DNA amplification. The obtained amplifiers were subjected to overnight restriction enzyme digestion followed by separation of the digested products on an agarose gel electrophoresis for detecting the presence or absence of specific point mutations. Initially all samples were examined for the 563C-T mutation of the Med- allele. Those remaining uncharacterized were analysed for 1003 A-G (Chatham) and 131 C-G (Orissa) [9]. The primers and restriction enzymes were utilized according to previously established protocols.

### Data analysis

After correction and coding, the complete dataset was double entered by two data entry operators using Epi-info, version 6.04. The two datasets were compared for data consistency and for missing values. Analysis was done by using STATA v8. The primary outcome is the proportion of people in the sample with phenotypic G6PD deficiency, as measured by the fluorescent spot test. The results were also stratified by the explanatory variables of ethnic group, gender and province (study site). Additional analysis was conducted on the genetic data to identify the proportion of males who were hemizygous deficient and the proportion of females who were homozygous deficient, non-deficient (wild-type) and heterozygous deficient, cross-tabulated against the FST result.

## Results

### Sample characteristics

In total, 1,500 individuals were enrolled in the study during July and August 2010. Data from 64 (4.3%) were excluded from the analysis for missing data (one missed the G6PD test result, 45 missed gender, ten missed ethnicity data, eight had age inaccurately recorded). Data from 1,436 participants were evaluated.

The sample characteristics are shown in Table 2. Mean age was 23 years, and 45% of the population was under 15 years, matching the age demographic for Afghanistan. Half of the sample was male.

**Table 2:** Characteristics of the sample

Total evaluable participants	1,436
Mean age	23.0 years
% age <15 years	45.7
% male	50.7
Ethnic group, n (%):	
*Pashtun*	519 (36.1)
*Tajik*	490 (34.1)
*Uzbek*	238 (16.6)
*Hazara*	152 (10.6)
*Turkmen*	2 (0.1)
*Arab*	10 (0.7)
*Pasheaa*	21 (1.5)
*Noristani*	4 (0.3)
Province (city name), n (%):	
*Bamyan*	89 (6.2)
*Faryab (Maimana)*	180 (12.5)
*Kabul*	688 (47.9)
*Nangahar (Jalalabad)*	295 (20.5)
*Takhar (Taloquan)*	184 (12.8)

All of the majority ethnic groups are included in the sample and most of the minority ethnic groups were also identified. For the main analysis the minority ethnic groups of Turkmen (n=2), Arab (n=10), Pashaee (n=22) and Noristani (n=4) have been grouped together because too few were in the sample for adequate analysis. The proportion of participants in each ethnic group does not appear to differ from widely available estimates of the ethno-linguistic composition of the country (e.g. [10]). The proportion of participants from each of the five provinces matches the relative size of the total provincial population.

### G6PD deficiency prevalence

Amongst the sample, 45 (3.1%) participants were phenotypically deficient for G6PD. Because the deficiency is X-linked, males and females were analysed separately. G6PD deficiency was found in 36/728 (5.0%) of males and 9/708 (1.3%) of females (chi^2^=16.0, p<0.001).

Prevalence of deficiency differed by ethnic group. Amongst both males and females G6PD deficiency was more frequent in Pashtuns than in other ethnic groups, and in Pashtun males deficiency was 10% (26/260) (Table 3).

**Table 3:** Prevalence of G6PD deficiency amongst males and females in different ethnic groups of Afghanistan [n/N (%)]

**Ethnicity**	Males*	**Females**
*Pashtun*	26/260 (10.0%)	6/257 (2.3%)
*Tajik*	8/244 (3.2%)	2/244 (0.8%)
*Uzbek*	0/125	0/113
*Hazara*	1/79 (1.3%)	1/73 (1.4%)
Other**	2/37 (5.4%)	1/20 (5.0%)
Total	35/724 (5.0%)	10/712 (1.3%)

* Chi^2^=24.5, p<0.001 (4d.f.); **Turkmen, Arab, Pashaee, Nooristani.

The frequency of G6PD deficiency did not differ by age group, as expected from a genetically inherited trait, but it did differ by province due to the differing ethnic make-up of each province.

The proportion of respondents who had a self-reported history of jaundice was higher amongst G6PD-deficient individuals than non-deficient individuals (7/45 [15.6%] *vs* 88/1,391 [6.3%]) (Fisher's exact test, p=0.038). There was no difference in the proportion of deficient or non-deficient participants who had had an anti-malarial, had self-reported malaria or who had ever been admitted to hospital.

Mutational analysis was successfully conducted on 480/500 subjects as follows: 44/45 samples identified as G6PD deficient by the fluorescent spot test [34M/10F], a random selection of 378 G6PD normal females and 67 G6PD normal males.

Table 4 shows the number and proportion of participants identified by genetic analysis and the fluorescent spot test. Sensitivity and specificity of the fluorescent spot-test among specimens from males was 89.2 and 98.4%, respectively. For females, in differentiation of homozygous from wild type, the sensitivity and specificity of the fluorescent spot test was 72.7 and 99.7%. Only one of the 34 heterozygous females was detected by the fluorescent spot test.

**Table 4:** ***G6PD c.563 C>T *variants in 44 G6PD-deficient and 436 non-deficient subjects**

	**Genetic testing results (PCR)**				
	**Male**		**Female**		
**FST result**	**haemizygous deficient**	**Wild type**	**Homozygous**	**Heterozygous**	**Wild type**
Non-deficient	4 (10.8)	60	3 (27.2)	33	336
Deficient	33	1 (1.7)	8	1	1 (0.3)

## Conclusions

G6PD deficiency is prevalent in the Afghan population and differs between ethnic groups. In male Pashtuns the prevalence of the deficiency is 10% and amongst female Pashtuns, 2%. Other ethnic groups also showed an appreciable prevalence.

The findings in this study are consistent with other studies in the region. In one such study [6] in Pashtun Afghan refugees in Pakistan, the prevalence of G6PD deficiency was about 10% amongst males. Bouma *et al.*[11] found that the prevalence of G6PD deficiency varied within the Pashtun population; amongst male Afghan pashtuns, prevalence was 15.8% while amongst male Pakistani pashtuns it was less (7%), although there was no statistical difference between the two groups. Any differences could be explained by the existence of many subgroups of Pashtun tribes [12] with intra-ethnic variance amongst these subgroups [13]. This may explain why Ali *et al.*[13] found a lower G6PD prevalence of 3.2% in ethnic Pashtun males in Pakistan.

Rehbolz *et al.*[14] found a prevalence of 2.1% in Tajikistan, and 1.4% amongst males of the Tajik ethnic group, consistent with the prevalence found in this study. The findings greatly differ for the Uzbek population, however. This study found zero amongst 238 tested ethnic Uzbek participants, where the study in Tajikistan [14] identified a prevalence of 4.2% and Bouma *et al.* found a prevalence of 9.1% among male Afghan refugees of Uzbek ethnicity in Pakistan [11]. This may be due to differences in reporting of ethnicity by the population under study as the parental lineage may be mixed.

The genetic analysis confirmed that almost all deficiency in this region is caused by the Med- allele. This variant has been associated with moderate to severe haemolytic reactions to primaquine. Testing for G6PD deficiency that is cheap and reliable is therefore required. Although one study indicates that the risk of malaria infection is lower in G6PD-deficient individuals [6] with an 85% protective effect, it is likely that around 1 or 2% patients will be deficient. The risk of exposing G6PD-deficient patients to severe complications may be lower than assumed but, crucially, is not zero.

The choice of test is an important factor and far from straightforward. The inaccuracy of the FST in this study in male patients could lead to false “normal” results. Detection of deficiency in females is difficult because female heterozygotes exhibit mozaicism and varying proportions of G6PD-deficient and non-deficient red cells circulate concurrently. Detecting heterozygous females is not possible with the phenotypic FST unless the patient is significantly deficient. The clinical implications of this are not clear [15]. A different cytochemical test may be employed for female patients, but is more expensive that the simple FST and requires more sophisticated laboratories. Prototype rapid tests show promise but provided few, though significant, false “normal” results [16].

The implications of an accurate and reliable test being widely used to improve radical cure are clear. The hypnozoite reservoir provides the means for the disease to transmit across the seasons – a peak of relapse episodes in the early season coincides with the bloom of mosquito vectors which is a characteristic of long latency subtropical strains of vivax. Relapses also render control interventions aimed at reducing vector-human contact less effective because episodes occur without the bite of a vector [17]. If the majority of patients could be treated with primaquine then the effect on transmission in areas of seasonal malaria could be dramatic. In G6PD-normal Afghan Pashtuns with vivax malaria in Pakistan who were treated with 14-day primaquine in a randomized trial, 2% of patients had one subsequent episode of vivax over 11 months of follow-up, while in the untreated group it was 30% [18,19]

Routine use of primaquine, as recommended in WHO and local guidelines, in all vivax patients would be a valuable control and elimination tool if applied to a high proportion of the population but this strategy requires accurate and reliable G6PD testing to be routinely available. If successfully employed, routine G6PD testing could result in significant reductions in transmission of vivax malaria.

## Competing interests

The authors declare that they have no competing interests.

## Authors’ contributions

TL, NM, HR, MS, OA and SJ designed the study and conducted the field work; TL conceived the study; TL and NM analysed the data; TL, NM and MV wrote the paper; BM, AM and AB conducted the genetic analysis and contributed to the paper. All authors provided intellectual input into this paper and read and approved the final version.
